# The Association between Vitamin D Deficiency and Sleep Disorders: A Systematic Review and Meta-Analysis

**DOI:** 10.3390/nu10101395

**Published:** 2018-10-01

**Authors:** Qi Gao, Tingyan Kou, Bin Zhuang, Yangyang Ren, Xue Dong, Qiuzhen Wang

**Affiliations:** 1 Department of the College of Public Health, Qingdao University, 38 Dengzhou Road, Qingdao 266021, China; gqi6835@163.com (Q.G.); tingyankou@sina.com (T.K.); 18363992818@163.com (Y.R.); dx18363997847@163.com (X.D.); 2 Department of the medical college of Qingdao University, Qingdao 266021, China; zhuangbin2762@163.com

**Keywords:** vitamin D, serum 25(OH)D, sleep, sleep quality, sleep duration, sleep disorders

## Abstract

Epidemiology studies have investigated the association between vitamin D and the risk of sleep disorders, but the results remain controversial. Therefore, we conducted this meta-analysis with the goal of clarifying the association between vitamin D and sleep disorders risk. All relevant studies were searched using PubMed, EMBASE, and Web of Science from inception to January 2018. Pooled odds ratios (ORs) and 95% confidence interval (CIs) were calculated using a fixed-effect model A total of nine studies (6 cross-sectional, 2 case-control, and 1 cohort studies) involving 9397 participants were included. By comparing the lowest verse highest levels of serum vitamin D, we found that participants with vitamin D deficiency (VDD) had a significantly increased risk of sleep disorders (OR: 1.50, 95% CI: 1.31, 1.72). Subgroup analysis showed that VDD also was associated with poor sleep quality (OR: 1.59, 95% CI: 1.23, 2.05), short sleep duration (OR: 1.74, 95% CI: 1.30, 2.32), and sleepiness (OR: 1.36, 95% CI: 1.12, 1.65). Subgroup analyses further indicated that serum 25(OH)D <20 ng/mL could significantly increase the risk of unhealthy sleep. This meta-analysis suggest that vitamin D deficiency is associated with a higher risk of sleep disorders. More high-quality cohort studies and randomized controlled trials (RCTs) are needed to verify this association.

## 1. Introduction

Sleep, which accounts for one-third part of the lifetime, is of the great essence in our daily routine [1]. The National Sleep Foundation recommends that adults should get 7–8 h sleep every day, albeit sleep demands may vary in age and gender [2]. As a modifiable lifestyle, healthy sleep is necessary for maintaining physical and psychological health. The daily sleep-wake cycle is controlled by circadian clock, different neurons, and hormones produced by the hypothalamus and environmental signals (dark/light) [3,4]. In recent years, sleep disorders have become an epidemic throughout the world [5,6,7,8], while many people, even medical staff, are not aware of their significance. Previous studies revealed that excessive sleep or sleep deprivation were associated with increased risk of adverse health events, including type II diabetes, hypertension, cancers, and all-cause mortality [9,10,11].

Vitamin D as a unique, fat-soluble vitamin can either be ingested from diet or synthesized by ultraviolet-B (UVB) radiation [12,13]. 25-hydroxyvitamin D (25(OH)D) is commonly considered as the best indicator of vitamin D status in the body [14]. Due to the concentrations of serum 25(OH)D can be affected by many factors (such as lack of sunlight exposure, lifestyle, and skin color), and vitamin D deficiency (VDD) is prevalent; lesser known functions of vitamin D are being paid more attention such as the association between VDD and cardiovascular diseases [15], infectious diseases [16], and sleep disorders [17]. Several studies reported that vitamin D receptors (VDR) were expressed in brain areas that regulate the sleep–wake cycle, such as the hypothalamus [18,19]. This evidence indicated that higher vitamin D status was inversely associated with the risk of sleep disorders.

Furthermore, several observational studies showed the association between vitamin D deficiency and sleep disorders. A cross-sectional study reported that vitamin D deficiency correlated with poorer sleep quality [20]. McCarty et al. observed that patients who exhibited vitamin D deficiency got lower scores on the Epworth Sleepiness Scale (ESSs) [21], which is an effective instrument for measuring excessive daytime sleepiness [22]. Additionally, several large sample epidemiology studies found that dietary intake of vitamin D was related to midpoint of sleep, sleep duration, and maintaining sleep [23,24,25]. However, the results were inconsistent. Gunduz et al. [26] found no significant difference in the Pittsburgh Sleep Quality Index (PSQI) total score between the VD-deficient group and the VD non-deficient group among women in the last trimester of pregnancy. The PSQI is a standardized self-questionnaire that measures sleep quality and disorders over a 1-month period [27].

To address the divergence mentioned above, we conducted this systematic review and meta-analysis to explore the association between vitamin D deficiency and the risk of sleep disorders.

## 2. Materials and Methods

### 2.1. Search Strategy

After identifying the study questions, we conducted this systematic review and meta-analysis following the Preferred Reporting Items for Systematic Reviews and Meta-Analyses (PRISMA) statement and Meta-analysis of Observational Studies in Epidemiology (MOOSE) guidelines. We carried out a systematic literature search from inception to the January 2018 in three databases (PubMed, EMBASE, and Web of Science) to identify relevant available articles reporting the relationship between vitamin D and sleep disorders. We used extensive search terms, and the complete PubMed search strategy is listed in Table 1. Titles and abstracts were examined to extract potentially relevant articles, followed by a more in-depth study of inclusion/exclusion criteria as described below in the literature results and research methods. Moreover, to find additional articles, we analyzed reference lists from relevance original and review articles by manual method.

### 2.2. Study Selection

Relevant studies were obtained and included if they (1) were cross-sectional, case-controlled, cohort studies; (2) evaluated the relationship between vitamin D deficiency and risk of unhealthy sleep; (3) provided odd ratios (ORs) or risk ratios (RRs) with 95% confidence intervals (CIs) or enough data to calculate these numbers; (4) provided the level of serum 25(OH)D. Meanwhile, we excluded studies if they (1) were cases, letters, editorials, or systemic reviews; (2) were on subjects with obstructive sleep apnea (OSA); (3) were not English language; (4) were non-human.

### 2.3. Data Extraction and Quality Assessment

Two authors (G.Q. and K.T.Y.) independently assessed the articles and extracted the data and disagreements were resolved through discussion. Extracted Information included: (1) the last name of the first author, publication year, and country; (2) study design details; (3) study population characteristics (number, age, and % female); (4) the methods of sleep measurement; (5) the cut-off value of vitamin D deficiency; (6) vitamin D measurement; and (7) adjusted confounding factors.

We evaluated the methodological quality of literature according to the 9-point Newcastle-Ottawa Quality Assessment Scale (NOS) [28]. This scale is composed of eight items in three parameters: (1) selection of cases and controls, (2) comparability of cases and controls, and (3) assessment of outcome. We defined studies that scored greater than 7 as high-quality.

### 2.4. Statistical Analysis

The OR with corresponding 95% CIs were used to measure the strength of the association between vitamin D deficiency and sleep disorders. If the adjusted effect OR and 95% CI were not reported, we constructed 2 *×* 2 tables (low vitamin D status versus presence or absence of sleep disorders) by extracting data from selected articles. Pooled estimates and corresponding 95% CIs were represented by forest plots.

Statistical heterogeneity across studies was evaluated with Cochrane Q test (*p* < 0.1 was considered significant) [29]. The *I*^2^ statistic was also calculated with the following cutoffs to evaluate heterogeneity: *I*^2^ = 0–25%, no heterogeneity; *I*^2^ = 25–50%, moderate heterogeneity; *I*^2^ = 50–75%, large heterogeneity; and *I*^2^ = 75–100%, extreme heterogeneity. If the results were no heterogeneity or moderate heterogeneity (*I*^2^ < 50%) [29], a fixed-effects model was used; otherwise, a random effect model was applied.

Subgroup analysis was performed to explore the potential source of heterogeneity. It was based on unhealthy sleep types, study design, location of study, number of participants, the cut-off of vitamin D, and season of blood sampling need.

To test the robustness of results, a sensitivity analysis was conducted by removing each study at a time. Small study publication bias was evaluated by using funnel plots, Egger’s tests, and Begg’s tests [30], in which *p* less than 0.05 suggested the exist of publication bias. All statistical analyses were performed using Stata 11.0 (Stata Corp., College Station, TX, USA).

## 3. Results

### 3.1. Literature Search and Study Characteristics

**Figure 1:**
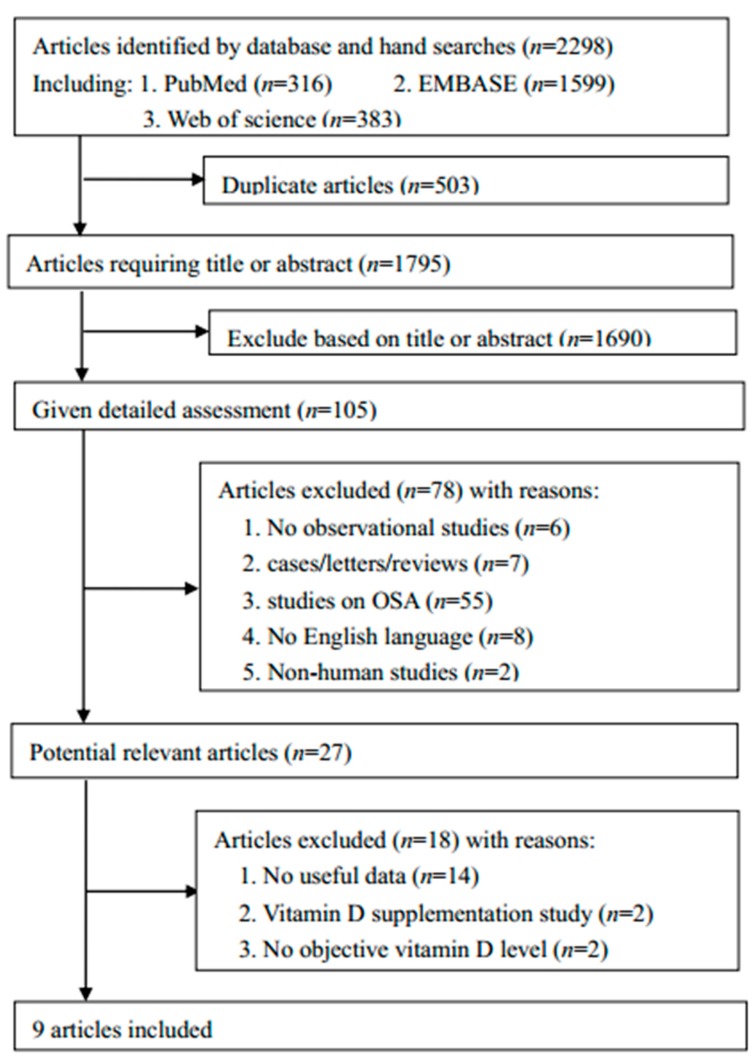
The flow chart of the selection of studies eligible for our meta-analysis.

For this meta-analysis, we searched a total of 2298 articles, after removing duplicates (*n* = 503), 1690 articles were excluded after reviewing the titles and abstract. Then, 105 articles were given detailed assessment, and the remaining 27 articles were screened for eligibility according to the full text. As a result, 9 studies were included in our meta-analysis. The detailed study selection is described in Figure 1 [20,26,31,32,33,34,35,36,37].

The basic characteristics of the included studies were summarized in Table 2. All studies were observational, among the nine studies, most were cross-sectional studies; two were case-control studies and only one was cohort study. Study populations ranged from 63 to 3048 with a total number of 9397 participants. Two studies were conducted in United States [20,31], two in France [32,34], and the rest in other countries. Most studies were mixed gender, two included only women [26,33], and one was only male [20]. For vitamin D deficiency cut-off value, the included studies mainly used 20 ng/mL [20,26,31,33,35] in accordance with the criteria suggested by Institute of Medicine (IOM) [38]; three studies used 30 ng/mL [32,34,37], and only one study used 10 ng/mL [36] as the cut-off value. Seven of the nine studies adjusted for potential confounding factors, whereas other studies provided only crude-effect estimates.

### 3.2. Meta-Analysis Result

**Figure 2:**
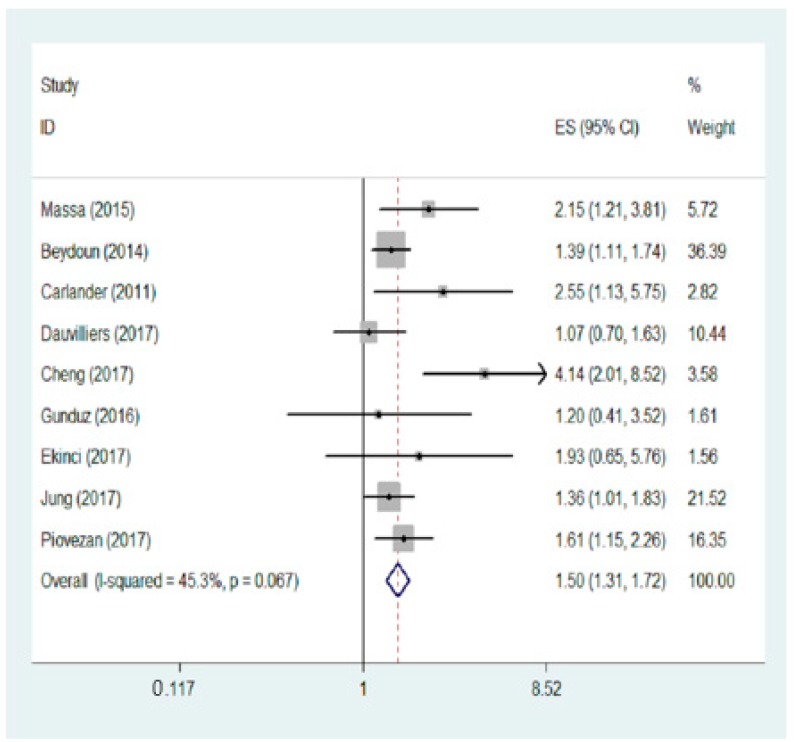
Forest plot for meta-analysis on the association between vitamin D and sleep disorders risk. Data showed low vs. high levels of serum vitamin D, using a fixed-effects model. ID: identification; ES: effect size.

There was moderate heterogeneity across the included studies (*p* < 0.001, *I*^2^ = 45.3%), so the fixed-effect model was used. The summary OR combining 9 studies showed that individuals with vitamin D deficiency had an increased risk of sleep disorders (OR: 1.50, 95% CI: 1.31, 1.72, *p* < 0.001) compared with high vitamin D (Figure 2).

### 3.3. Subgroup Analysis

**Figure 3:**
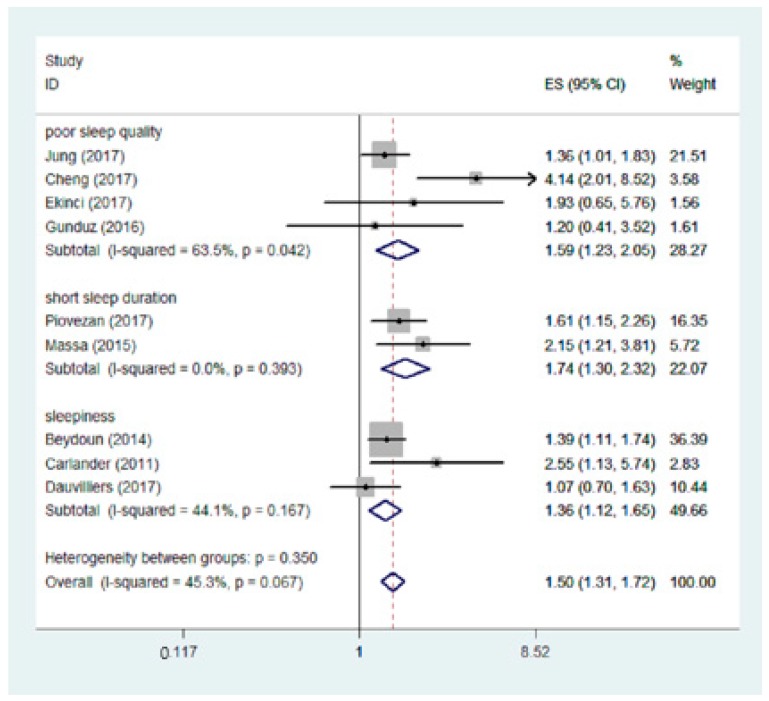
Subgroup analysis of the association between vitamin D (lowest vs. highest) and the risk of three kinds of sleep disorders. ID: identification; ES: effect size.

We performed a series of subgroup analyses to explore the source of heterogeneity (Table 3). We stratified the included studies according to study design, sample size, sleep characteristics, study region, and the cut-off value of vitamin D deficiency. The pooled OR of the six cross-sectional studies was 1.47 (95% CI: 1.27, 1.71). The subgroup analyses also showed a more significant risk of sleep disorders with serum 25(OH)D levels ≤ 20 ng/mL (OR: 1.59, 95% CI: 1.31, 1.94). In addition, we separated poor sleep quality, short sleep duration, and sleepiness as 3 different outcomes to conduct subgroup analysis. The results were almost the same as the overall sleep disorders (Figure 3).

### 3.4. Sensitivity Analysis

To prove the robustness of the results, we performed the sensitivity analyses by excluding a class of study at a time (Table 4). In the sensitivity analysis, the estimate effects (OR) with 95% CI ranged from 1.41 (1.22, 1.63) to 1.63 (1.28, 2.07). All values conferred *p* ≤ 0.001. The sensitivity analyses showed that removing any single study from the entire sample had little substantially influence in the estimate effect (OR with 95% CI), indicating that our results were robust.

### 3.5. Publication Bias

**Figure 4:**
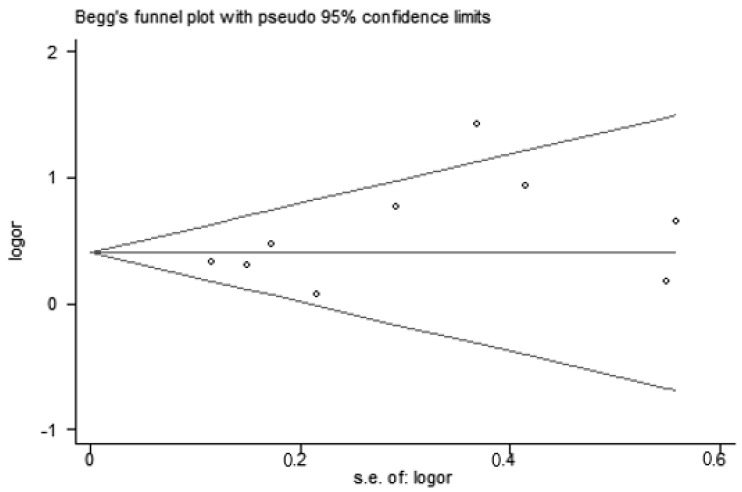
The funnel plots of vitamin D deficiency and the risk of sleep disorders.

Using the funnel plots, Begg’s test, and Egger’s test (Figure 4), we established that there was no publication bias, because symmetrical distributions of studies were on both sides of the average. Results from Begg’s and Egger’s tests also did not reveal any evidence of publication bias (Begg’s *p* = 0.17, Egger’s *p* = 0.14).

## 4. Discussion 

This meta-analysis discussed the relationship between vitamin D deficiency and sleep disorders including poor sleep quality, short sleep duration, and sleepiness. Overall, the results showed that serum 25(OH)D levels were inversely associated with an increased risk of sleep disorders.

Our finding indicates that low serum 25(OH)D may be a risk factor of unhealthy sleep (OR: 1.50, 95% CI: 1.31, 1.72). In subgroup analysis, sleep disorders were divided into three types in order to improve the generality of the results. There was significant inverse association between vitamin D status and the risk of short sleep duration. In addition, we used 10, 20, and 30 ng/mL cut-offs value in subgroup analysis because of the discrepancy in the standard of vitamin D deficiency [38,39]. The results showed that the cut-off value of 20 ng/mL, in accordance with the criteria suggested by IOM, increased the risk of poor sleep quality by nearly 60% (pooled OR: 1.59, 95% CI: 1.31, 1.94). Our results further indicate that this criterion is more suitable for studying the correlation of vitamin D deficiency and the risk of certain chronic diseases and life quality.

The sensitivity analysis results showed that two studies may contribute to heterogeneity [26,33]. There was a low degree of heterogeneity after excluding these two articles. Heterogeneity may be caused by pregnant women’s decision to act responsibly, which affects their dietary and sleep behavior during pregnancy. Results in the sensitivity analysis ranged from 1.41 (1.22, 1.63) to 1.63 (1.28, 2.07) showed that our results were robust. Based on the funnel plot, there is no publication bias. However, due to the small number of included studies, it is difficult to say that there is no asymmetry in the plot. Hence, we further evaluated the publication bias by Begg’s and Egger’s tests.

In addition to bone homeostasis, vitamin D plays a role in multiple physiological mechanisms, including sleep, immunity, and others [40]. Although the underlying mechanisms to explain the association between vitamin D deficiency (VDD) and sleep disorders are not yet known, several possible mechanisms have been suggested. Recent experimental studies have identified that vitamin D receptors (VDR) are common in nearly all tissues of the body, including the central nervous system [41]. VDR are widely distributed in human brain, such as the hypothalamus, prefrontal cortex, midbrain central gray, substantia nigra, and raphe nuclei, all of which are known to execute important roles in sleep regulation [42,43]. VDD [44] is a prevalent condition that is associated with a deforming demineralization of bones, as well as more recent problems such as chronic nonspecific pain [45,46,47,48,49,50], which may cause poor sleep [51,52]. Chronic, nonspecific clinical pain seems to be a marker of VDD: The prevalence of VDD was high in patients who complained about intractable pain of an uncertain cause. Okura, et al. reported that individuals with chronic pain gained an increased risk of poor sleep quality and short sleep duration [52]. Increased pain sensation related to sleep deprivation is reported to be associated with an increase in IL-6 [53]; an inflammatory marker can be elevated in patients with obstructive sleep apnea (OSA) [54] and low 25(OH)D [55]. One study about veterans with chronic pain found that their pain levels, sleep quality, and various aspects of Quality of Life (QoL) can be significant improved after standardized vitamin D supplementation [56]. Furthermore, Vitamin D deficiency increases the risk of autoimmune disease and respiratory infectious diseases. Emerging lines of evidence suggest that vitamin D can play an immunomodulatory role by altering immune regulation, decreasing the release of inflammatory substances, including those that regulate sleep, such as prostaglandin D2, tumor necrosis factor alpha (TNF-a), and cytokine [57,58,59]. Barcelo et al. founded that patients with obstructive sleep apnea syndrome (OSAS) had higher levels of lipocalin-type PGD synthase than patients without OSAS [60]. In summary, recent articles suggest that VDD regulates the development of symptoms of wakefulness that are commonly related to sleep disorders [21]. There remains much to be studied about the complex relationship between long-term low levels of vitamin D, normal sleep, and sleep disorders.

Moreover, some epidemiologic evidence has verified the impact of vitamin D supplements on sleep disorders. An intervention study reported that vitamin D supplementation (D3) in veterans (50,000 IU/week) increased their sleep duration [56]. Another double-blind clinical trial showed use of vitamin D supplementation (50,000 IU/fortnight for 8 weeks) facilitated sleep duration and quality in people with sleep disorder [61]. Consequently, combined with our findings, it may be useful to ameliorate poor sleep by increasing vitamin D levels in the body. However, as there were only two reported RCT studies and the existing high heterogeneity, we cannot include them in this meta-analysis.

The present meta-analysis has several strengths. First, to our knowledge, this is the first meta-analysis to assess the association between VDD and sleep disorders. Second, we use poor sleep quality, short sleep duration, and sleepiness as three different outcomes to conduct subgroup analysis. The results were almost as same as the overall sleep disorders. Furthermore, the subgroup and sensitivity analysis validated the reliability and robustness of our results.

Several potential limitations of this meta-analysis should be recognized. First, the number of studies eligible for our meta-analysis was small, and different study designs also prevented data extraction. Second, heterogeneity that existed in the studies should not be ignored when we interpret the results. The heterogeneity may be due to the difference in the assessment of sleep and grouping criteria of vitamin D. Last, most of the studies were cross-sectional, so they cannot establish a causal association. Therefore, further high-quality cohort studies and well-designed randomized controlled trials (RCTs) are needed to verify this relationship and to determine the effect of vitamin D supplementation in unhealthy sleep therapy.

## 5. Conclusions

In conclusion, this meta-analysis demonstrates that vitamin D deficiency is associated with a higher risk of sleep disorders in the population. Subgroup analyses further indicated that vitamin D < 20 ng/mL could increase the risk of sleep disorders. More high-quality and well-designed randomized controlled trials (RCTs) should be conducted to confirm the role of vitamin D supplementation in the prevention and therapy of sleep disorders.

## Figures and Tables

**Table 1:** The search strategy in PubMed of the relationship between vitamin D and sleep disorders.

Search Terms
#1 sleep [Mesh Terms]
#2 sleep duration * OR sleep quality * OR sleep disorders * OR short sleep OR hypersomnia OR sleep OR sleep time OR Short-term sleep restriction OR daytime sleepiness OR long sleepers OR short sleepers OR sleep initiation and maintenance disorders OR habitual short sleepers OR sleep deprivation OR nap OR napping OR sleep disturbance OR sleep disorders OR siesta OR sleep time OR drowse OR insomnia OR drowsiness OR 24-h sleep duration OR night time sleep duration OR short sleep duration OR long sleep duration
#3 #1 OR #2
#4 vitamin D [Mesh Terms]
#5 vitamin D analogues OR doxercalciferol OR alfacalcidol OR vitamin D3 OR vitamin D2 OR activated vitamin D OR 1alpha-vitamin D OR calcitriol OR calcidiol OR 1,25dihydroxycholecalciferol OR 25-hydroxyvitamin D2 OR calcifediol OR 1,25OH2D OR dihydrotachysterol OR ergocalciferols OR 25OHD OR Vit D OR 25-hydroxy vitamin D2 OR VitD OR vitamin D-3 OR 25-hydroxycholecalciferol OR 25OHD OR 25-hydroxy-vitamin D OR ergocalciferol OR 1,25-dihydroxyvitamin D3 OR 25-OH vitamin D OR cholecalciferol OR 25-hydroxyvitamin D
#6 #4 OR #5
#7 #3 and #6

**Table 2:** Characteristics of studies reporting the association between vitamin D deficiency and sleep disorders.

Author, Year, Country	Study Design	Sample Size (Age;% Female)	Sleep Measurement	25(OH)D Cutoffs ng/mL	Sleep Characteristic	Vitamin D Measurement	Adjusted Variable	NOS
Cheng, 2017 [33], Singapore	cohort	1152 (≥18, 100%)	PSQI	<20	Sleep quality	ID-LC–MS/MS	ethnicity, age, early pregnancy BMI, education, household income, parity, night shift, status, physical activity, total EPDS score, and gestational weight gain per week.	9
				20–32				
				>32				
Ekinci, 2017 [35], Turkey	cross-sectional	63 (3–16; 47.6%)	PSQI	<20	Sleep quality	HPLC	NO	7
				≥20				
Gunduz, 2016 [26], Turkey	cross-sectional	92 (18–45; 100%)	PSQI	<20	Sleep quality	HPLC	NO	6
				20–32				
				>32				
Jung, 2017 [36], Korea	cross-sectional	1472 (19–39; 20%)	PSQI	<10	Sleep quality	ECLIA	age, sex, marital status, level of education, BMI, smoking habits, alcohol consumption habits, regular exercise, employee tenure, occupational stress	7
				>10				
Massa, 2015 [20], USA	cross-sectional	3048 (≥65; 0%)	wrist actigraphy	<20	Sleep duration	LC-MS/MS	age, clinic, season, comorbidities, BMI, physical and cognitive function.	7
				20–30				
				30–40				
				≥40				
Piovezan, 2017 [37], Brazil	cross-sectional	657 (28–78; 56%)	polysomnography	<30	Sleep duration	CMIA	age, gender, ethnicity, obesity, smoking, hypertension, diabetes, sedentary lifestyle, seasonality, creatinine serum levels	7
				>30				
Beydoun, 2014 [31], USA	cross-sectional	2459 (20–80; 52%)	sleep questionnaire	<20	Sleepiness	HPLC	age, sex, race/ethnicity, education, marital status, and family income	6
				≥20				
Carlander, 2011 [32], France	case-control	106 (16–65; 60%)	poly-somnography ESS	<30	Sleepiness (NC)	RIA	age at onset, duration and severity of disease at baseline, treatment intake at time of study, season of blood sampling	6
				>30				
Dauvilliers, 2017 [34], France	case-control	348 (6–68; 35%)	ESS; AESS	<30	Sleepiness (NC)	RIA	age, BMI, and season of blood sampling	8
				>30				

BMI: Body Mass Index; EPDS: Edinburgh Postnatal Depression Scale; PSQI: Pittsburgh sleep quality index; ESS: Epworth Sleepiness Scale; AESS: Adapted Epworth Sleepiness Scale; NC: Narcolepsy with cataplexy; ECLIA: electrochemiluminescence immunoassay; ID-LC–MS/MS: isotope dilution liquid chromatography–tandem mass spectrometry; CMIA: chemiluminescent microparticle immunoassay; HPLC: high performance liquid chromatography; LC-MS/MS: liquid chromatographytandem mass spectrometry; RIA: radioimmunoassay; NOS: Newcastle-Ottawa Quality Assessment Scale.

**Table 3:** Pooled estimates for vitamin D status (lowest vs. highest) and risk of sleep disorders in subgroups of trials.

Group		Number	OR	95% CI	*p* Value	*I*^2^ (%)
All		9	1.50	1.31, 1.72	<0.001	45.3%
Study design	cross-sectional	6	1.47	1.27, 1.71	<0.001	0.0%
	cohort	1	4.14	2.01, 8.52	0.02	
	case-control	2	1.29	0.89, 1.87	0.18	71.1%
Sample size	<1000	5	1.46	1.15, 1.86	0.002	12.9%
	≥1000	4	1.52	1.29, 1.79	<0.001	69.9%
Sleep characteristic	Poor sleep quality	4	1.59	1.23,2.05	<0.001	63.5%
	Short sleep duration	2	1.74	1.30, 2.32	<0.001	0.0%
	Sleepiness	3	1.36	1.12, 1.65	0.002	44.1%
Vitamin D cut off	10 ng/mL	1	1.36	1.01, 1.83	0.04	
	20 ng/mL	5	1.59	1.31, 1.94	<0.001	58.2%
	30 ng/mL	4	1.46	1.13, 1.87	0.003	52.5%
Geographic location	Asia	4	1.59	1.23, 2.05	<0.001	63.5%
	European	2	1.51	1.26, 1.81	0.18	71.0%
	America	3	1.29	0.89, 1.87	<0.001	5.8%

OR: odds ratio; CI: confidence interval; *I*^2^: inconsistency.

**Table 4:** Pooled estimates for vitamin D status and risk of sleep disorders for low vs. high quantile through sensitivity analyses.

Group	Number	OR	95% CI	*p*	*I*^2^ (%)
All	9	1.50	1.31, 1.72	<0.001	45.3%
Exclude unadjusted	7	1.63	1.28, 2.07	<0.001	57.9%
Exclude only men	8	1.47	1.28, 1.69	<0.001	46.2%
Exclude only women	7	1.45	1.26, 1.67	<0.001	9.4%

OR: odds ratio; CI: confidence interval; *I*^2^: inconsistency.
